# Blood manganese concentrations in Jamaican children with and without autism spectrum disorders

**DOI:** 10.1186/1476-069X-13-69

**Published:** 2014-08-23

**Authors:** Mohammad H Rahbar, Maureen Samms-Vaughan, Aisha S Dickerson, Katherine A Loveland, Manouchehr Ardjomand-Hessabi, Jan Bressler, Sydonnie Shakespeare-Pellington, Megan L Grove, Deborah A Pearson, Eric Boerwinkle

**Affiliations:** 1 Division of Epidemiology, Human Genetics, and Environmental Sciences (EHGES), University of Texas School of Public Health at Houston, Houston, Texas, USA; 2 Division of Clinical and Translational Sciences, Department of Internal Medicine, University of Texas Medical School at Houston, Houston, Texas 77030, USA; 3 Biostatistics/Epidemiology/Research Design (BERD) component, Center for Clinical and Translational Sciences (CCTS), University of Texas Health Science Center at Houston, UT Professional Building Suite 1100.05, 6410 Fannin Street, Houston, TX 77030, USA; 4 Department of Child & Adolescent Health, The University of the West Indies (UWI), Mona Campus, Kingston, Jamaica; 5 Department of Psychiatry and Behavioral Sciences, University of Texas Medical School at Houston, Houston, Texas 77054, USA; 6 Human Genetics Center, School of Public Health, University of Texas Health Science Center at Houston, Houston, Texas 77030, USA

**Keywords:** Manganese, Autism Spectrum Disorder, Neurodevelopment, Seafood, Vegetables, Jamaica

## Abstract

**Background:**

Manganese is an essential element for human health and development. Previous studies have shown neurotoxic effects in children exposed to higher levels of manganese. Autism Spectrum Disorder (ASD) is a neurodevelopmental disorder that impairs social interaction and communication. Several studies have hypothesized that ASD is caused through environmental exposures during crucial stages in brain development. We investigated the possible association between blood manganese concentrations (BMC) and ASD. We also identified factors associated with BMC in typically developing (TD) Jamaican children.

**Methods:**

We used data from 109 ASD cases with their 1:1 age- and sex-matched TD controls to compare mean BMC in Jamaican children (2–8 years of age) with and without ASD. We administered a pre-tested questionnaire to assess demographic and socioeconomic information, medical history, and potential exposure to manganese. Finally, we collected 2 mL of whole blood from each child for analysis of manganese levels. Using General Linear Models (GLM), we assessed the association between BMC and ASD status. Furthermore, we used two independent sample t-tests to identify factors associated with BMC in TD children.

**Results:**

In univariable GLM analysis, we found no significant association between BMC and ASD, (10.9 μg/L for cases vs. 10.5 μg/L for controls; *P* = 0.29). In a multivariable GLM adjusting for paternal age, parental education, place of child’s birth (Kingston parish), consumption of root vegetables, cabbage, saltwater fish, and cakes/buns, there was still no significant association between BMC and ASD status, (11.5 μg/L for cases vs. 11.9 μg/L for controls; *P* = 0.48). Our findings also indicated TD children who ate fresh water fish had a higher BMC than children who did not (11.0 μg/L vs. 9.9 μg/L; *P* = 0.03) as younger TD children (i.e., 2 ≤ age ≤4), (12.0 μg/L vs. 10.2 μg/L; *P* = 0.01).

**Conclusions:**

While these results cannot be used to assess early exposure at potentially more susceptible time period, our findings suggest that there is no significant association between manganese exposures and ASD case status in Jamaica. Our findings also indicate that BMC in Jamaican children resemble those of children in the developed world and are much lower than those in the developing countries.

## Background

Autism Spectrum Disorder (ASD) is a neurodevelopmental disorder that impairs social interaction and communication [1-3]. Currently, the etiology of ASD is not well understood. A number of studies have indicated that ASD has a heritable factor [4,5]. Others have suggested that there are combinations of factors that influence the etiology of ASD [6-8], including the interaction of genes with environmental factors [9]. Furthermore, studies have also hypothesized that ASD are caused through environmental exposures during crucial stages in brain development [10].

Manganese (Mn) is a naturally occurring trace element essential for human health and development [11,12]. On the other hand, excess exposure to manganese could cause neurotoxicity, which will be described in detail below [12-26]. Inorganic compounds, such as manganese phosphate, manganese sulfate, and manganese oxides, can be formed during combustion processes in coal-burning industrial facilities and automobile motors [11]. Exposure to these compounds through ambient air is of concern because there is greater neurotoxicity from inhaled manganese than from orally ingested manganese [27]. This is due to biological absorption though the lungs or olfactory epithelium, allowing manganese to bypass the liver and go directly to the brain [28]. Previous studies have reported that levels of manganese found in Jamaican soil are approximately twice the average levels reported for soils in other countries based on a world-wide comparison performed by the International Atomic Energy Agency [29]. Free Mn^2+^ ions are readily absorbed by plants and can be easily exchanged with Ca^2+^, making fruits, vegetables, and grains primary nutritional sources of manganese [12,30,31]. In Jamaica, the concentration of manganese in legumes has been reported, with the highest concentration in cowpeas [31]. On the other hand, manganese and other heavy metals can accumulate in tissues of fish and bottom-feeding mollusks, posing a risk to human health [32-34], with a higher risk in those residing in coastal communities where local fish consumption is greater [32-34].

Excess exposure or deficiency of manganese can cause harmful effects in children [24]. Intake of adequate amounts of manganese is usually maintained through a balanced diet; insufficient amounts can lead to poor developmental outcomes, including slowed growth [12,35,36]. This occurrence in children is very rare, however, the effects of deficient manganese exposure in humans are not well documented [37,38].

Children have a higher rate of absorption and/or retention of manganese compared to adults [12]. Manganese primarily targets the nervous system and has been shown to have adverse effects on neuromuscular [12] and cognitive function [13] in adults exposed to excessive amounts. Other studies have also shown adverse neurological effects in children exposed to higher levels of manganese including increased behavioral problems [14-16], reduced verbal and full-scale intelligence quotient (IQ) scores [17-21], diminished attention [22], and lower academic achievement [23,24]. In a study on children (12 months of age) from Mexico City, Claus Henn *et al*. [25] reported an inverted U-shaped association between blood manganese concentrations (BMC) and concurrent mental development scores [25] which supports a recent report that both low and high manganese levels may have adverse effects on neurodevelopment in early life [24].

Several studies have examined the relationship between ASD and manganese exposure as measured by air distribution [39], tooth enamel [40], hair [40-45], urine [44], and red blood cells [46], but their findings are conflicting. For instance, a case–control study of 84 children (ages 9–14 years) recruited from two multi-site national research studies in the US reported that mean manganese concentrations in postnatal regions of tooth enamel in children with ASD was lower in comparison to levels in primary teeth of typically developing (TD) children matched on gender, race, parents’ education, and parents’ marital status (1.87 ppm vs. 2.91 ppm, *P* = 0.08) [40]. In contrast, another case–control study of 44 children with ASD and 61 age-balanced controls showed no difference in the distributions of manganese in hair between ASD cases and TD controls (*P* = 0.84) [43].

As an island nation with a unique soil composition [29], Jamaica offers the opportunity to study effects of exposures to various environmental contaminants. In this study, we investigated the possible association between BMC and ASD status. As a second objective, we also identified factors associated with BMC in TD Jamaican children.

## Materials and methods

### General description

The Jamaican Autism study is an age- and sex-matched case–control study that began enrollment in December 2009, with an aim to investigate possible associations between ASD and postnatal environmental exposure to five heavy metals of interest: lead, mercury, arsenic, cadmium, and manganese. Details regarding recruitment and screening of children have been reported previously [47-50]. In summary, children listed in the University of West Indies’ (UWI) Jamaica Autism Database who were identified as being at risk for having an ASD based on assessment from trained clinicians using the Diagnostic and Statistical Manual of Mental Disorders-Text Revision (DSM-IV-TR) criteria [51] and the Childhood Autism Rating Scale (CARS) [52] were invited for reassessment of ASD. Parents who provided consent were administered the Autism Diagnostic Interview-Revised (ADI-R) [53] and their children underwent assessment using the Autism Diagnostic Observation Schedule (ADOS) [54] to confirm the previously suggested diagnosis using established algorithms and cut-off points for scoring the ADOS [55] and ADI-R [56]. Inclusion criteria for the study included birth in Jamaica and age between 2 and 8 years at enrollment. Age- and sex- matched TD controls from schools and well-child clinics were then identified for each confirmed ASD case. We also administered the Lifetime Form of the Social Communication Questionnaire (SCQ) [53] to parents or guardians of TD controls to screen for symptoms of ASD and included those with an SCQ score of 0–6, 6 being one standard deviation above the mean SCQ score of TD children [57].

Before enrollment, all parents and guardians provided written informed consent in compliance with the Institutional Review Boards of the University of Texas Health Science Center at Houston and UWI. We then administered pre-tested demographic and food frequency questionnaires to parents or guardians of all children participating in the study to gather information on socioeconomic (SES) characteristics and potential exposure to manganese through nutritional intake. Food frequency questionnaires asked how often the child ate certain foods each week, with a particular focus on types of fruits, vegetables, grains, and proteins [47,49,50]. We collected 2 mL of venous whole blood from each child at the end of each interview. These blood samples were frozen and stored at −20°C, then shipped to the Michigan Department of Community Health (MDCH) Trace Elements Laboratory for analysis of exposure to metals of interest. The data presented here are from the analysis of 218 children (109 ASD cases and 109 age- and sex-matched TD controls) that were evaluated between December 2009 and March 2012.

### Assessment of manganese exposure

Jamaican soils contain a unique distribution of a variety of trace elements, including manganese that can accumulate in crops and seafood [29,31]. Because the Jamaican population consumes large amounts of locally grown fruits and vegetables along with large amounts of seafood [31], the residents may have continuous exposure to manganese, resulting in bioaccumulation [58]. For this study, venous whole blood samples were diluted and analyzed using a PerkinElmer Elan DRCII inductively-coupled plasma mass spectrometer (PerkinElmer, Waltham, MA). Assays for blood manganese were performed at the MDCH Trace Metals Laboratory, a facility certified by the Centers for Disease Control and Prevention (CDC) for trace metal analyses. All BMC in our samples are above the limit of detection of 1 μg/L.

### Statistical analysis

We used descriptive analyses to compare demographic characteristics and SES of enrolled ASD cases and TD controls. Since the distribution of BMC was approximately normal, we did not transform the manganese data. Therefore, the means reported are arithmetic means. To account for the age- and sex-matched study design when comparing BMC of ASD cases and TD controls, we used General Linear Models (GLM), which are equivalent to standard linear regression models with normality assumption of the residuals but allow assessment of random effects. As described in our earlier reports, we further controlled for the effect of matching by using 108 dummy variables and one referent pair to represent the 109^th^ matched pair [47,49]. To avoid multicollinearity due to high correlation between maternal and paternal education levels, we combined these variables into one binary variable indicating if at least one parent had education beyond high school or both parents had education up to high school. To test associations between ASD case status and exposure variables we used Conditional Logistic Regression (CLR). We also used GLMs to evaluate the association between blood manganese concentrations and potential exposures to manganese, including frequency of consumption of various food items by children. Furthermore, we used t-tests to assess the same associations for ASD and TD control groups separately.

Lastly, we fit a multivariable GLM to investigate the relationship between ASD and BMC in children while adjusting for potential confounders. To identify possible confounders that were included in our final multivariable model, we determined variables to be potentially associated with ASD case status if *P* < 0.25 in the CLR model and potentially associated with BMC if *P* < 0.25 in the GLM. The covariates were considered to be potential confounders if they changed the regression coefficient by > 10%, and were then included in the final multivariable analysis [59]. We examined the role of potential confounders associated with various sources of manganese exposure including consumption of root vegetables, leafy vegetables, legumes, fruits, seafood, and grain products as well as drinking and cooking water source, parental ages, and parental education levels. Additionally, because a disproportionate number of our controls were drawn from the Kingston area and parental education level is a known factor associated with ASD, place of child’s birth (Kingston parish vs. other parishes) and parental education level were considered as *a priori* potential confounders in our multivariable model. Finally, we categorized the BMC into four quartiles and assessed the possible association between the categorized BMC variable and ASD status by univariable and multivariable CLR models and using the lowest 25% of BMC as the referent category. The purpose of these alternative analyses is to provide estimates for unadjusted and adjusted matched odds ratios that will allow comparisons of our findings with those of other case–control studies. All statistical analyses were conducted at the 0.05 level of significance using SAS 9.3® statistical software [60].

## Results

The mean age of children in our sample was approximately 67 months. Of the 218 children recruited, 184 (84.4%) were male. ASD cases and TD controls were 92.7% and 99.1% Afro-Caribbean, respectively. Maternal and paternal education levels beyond high school for both parents were significantly lower in TD controls compared to ASD cases (23.6% vs. 48.6% for mothers and 12.4% vs. 45.3% for fathers; *P* < 0.01). The percentage of TD controls born in Kingston parish was significantly greater than in other parishes (69.7% vs. 20.2%; *P* < 0.01). Additionally, compared to the ASD group, a greater percentage of families of the TD control group had three or more children in the household (46.8% of controls and 21.1% of cases; *P* < 0.01). The socioeconomic status of the ASD group, measured by reported possessions, was also higher than that of TD controls. Other demographic characteristics are displayed in Table 1.

**Table 1:** Demographic and socioeconomic characteristics of children and their parents by ASD status

**Variables**	**Categories**	**ASD Case (n = 109)**	**TD Control (n = 109)**	***P*-value**
		**N (%)**	**N (%)**	
**Sex of child**	Male	92 (84.4)	92 (84.4)	1.00
**Age of child (months)**	Age < 48	21 (19.3)	18 (16.5)	0.29
	48 ≤ age < 72	49 (45.0)	50 (45.9)	
	Age ≥ 72	39 (35.7)	41 (37.6)	
**Place of child’s birth**	Kingston parish	22 (20.2)	76 (69.7)	<0.01
	Other parishes	87 (79.8)	33 (30.3)	
**Maternal education **^***a***^**(at child’s birth)**	Up to high school^*^	56 (51.4)	81 (76.4)	<0.01
	Beyond high school^**^	53 (48.6)	25 (23.6)	
**Paternal education **^***b***^**(at child’s birth)**	Up to high school^*^	58 (54.7)	92 (87.6)	<0.01
	Beyond high school^**^	48 (45.3)	13 (12.4)	
**Number of children in the household (Age ≤ 18 years)**	1 - 2	86 (78.9)	58 (53.2)	<0.01
	≥ 3	23 (21.1)	51 (46.8)	
**Number of adults in the household (Age > 18 years)**^***c***^	1 - 2	72 (66.1)	65 (60.7)	0.47
	≥ 3	37 (33.9)	42 (39.3)	
**Assets owned**	TV	107 (98.2)	102 (93.6)	0.10
	Refrigerator	106 (97.2)	93 (85.3)	<0.01
	Freezer	14 (12.8)	21 (19.3)	0.20
	Living room set	91 (83.5)	51 (46.8)	<0.01
	Washing machine	79 (72.5)	57 (52.3)	<0.01
	Cars or other vehicle	72 (66.1)	39 (35.8)	<0.01
	Telephone/Cell phone	109 (99.1)	108 (99.1)	1.00
	Cable/Satellite connection	68 (62.4)	40 (36.7)	<0.01

^*^Up to high school education includes: attended primary, junior-secondary, and secondary/high/technical schools.

^**^Beyond high school education includes: attended vocational, tertiary college, or university.

^*a*^Maternal education was missing for 3 controls.

^*b*^Paternal education was missing for 3 cases and 4 controls.

^*c*^Number of adults in the household was missing for 2 control families.

Univariable analysis of the relationship between BMC and ASD case status showed no statistically significant difference between mean BMC in ASD cases compared to TD controls (10.9 μg/L for ASD cases vs. 10.5 μg/L for TD controls, *P* = 0.29). We used CLR to investigate potential associations between various sources of exposure and ASD status. Both maternal and paternal age were higher for ASD cases than TD controls [Matched Odds Ratio (MOR) = 3.13, 95% CI (1.41, 6.93) for maternal age and MOR = 2.54, 95% CI (1.34, 4.82) for paternal age]. Parental education levels were also significantly higher for ASD cases compared to TD controls [MOR = 3.36, 95% CI (1.85, 6.10)]. We also identified dietary intake differences between ASD cases and TD controls. For example, parents indicated that TD controls had a significantly higher consumption of fruits, vegetables, grains, and seafood than ASD cases, except for string beans, rice, shellfish, and shrimp as well as whole wheat bread, root vegetables (“yam, sweet potato, or dasheen”), and fresh water fish that were marginally significant. Associations between various exposure variables and ASD status are presented in Table 2.

**Table 2:** Association between potential confounders and ASD case status using Conditional Logistic Regression (CLR) (218 children, 109 matched-pairs)

**Variables**	**Category**		**ASD Case**	**TD Control**	**Matched OR**	**95% CI for MOR†**	***P*-value**
			**N (%)**	**N (%)**	**(MOR)**		
Paternal age^*a*^	More than 35 years		53 (50.5)	29 (29.0)	2.54	(1.34, 4.82)	<0.01
(at child’s birth)							
Maternal age^*b*^	More than 35 years		28 (25.7)	11 (10.6)	3.13	(1.41, 6.93)	<0.01
(at child’s birth)							
Parental education levels^*c*^	At least one of the parents had education beyond high school		71 (67.0)	32 (31.4)	3.36	(1.85, 6.10)	<0.01
(at child’s birth)							
Source of drinking water^*d*^	Piped water		103 (94.5)	104 (96.3)	0.60	(0.22, 1.65)	0.32
Source of water for cooking^*e*^	Piped water		103 (94.5)	103 (95.4)	0.83	(0.25, 2.73)	0.76
Fruits and vegetables consumption^*f*^	Root vegetables	A. Yam, sweet potato, or dasheen	78 (71.6)	89 (82.4)	0.52	(0.27, 1.02)	0.06
		B. Carrot or pumpkin	94 (86.2)	106 (98.1)	0.14	(0.03, 0.63)	0.01
	Leafy vegetables	A. Lettuce	51 (46.8)	67 (62.0)	0.59	(0.35,0.99)	0.04
		B. Callaloo, broccoli, or pakchoi	79 (71.6)	101 (93.5)	0.23	(0.10, 0.51)	< 0.01
		C. Cabbage	73 (67.0)	101 (93.5)	0.19	(0.09, 0.44)	< 0.01
	Legumes	String beans	36 (33.0)	45 (41.7)	0.71	(0.42, 1.20)	0.19
	Fruits	Tomatoes	66 (60.6)	90 (83.3)	0.29	(0.14, 0.58)	< 0.01
		Ackee	64 (58.7)	100 (92.6)	0.05	(0.01, 0.22)	< 0.01
		Avocado	30 (27.5)	73 (67.6)	0.17	(0.08, 0.35)	< 0.01
		Green banana	74 (67.9)	98 (90.7)	0.29	(0.14, 0.58)	< 0.01
		Fried plantains	78 (71.6)	96 (88.9)	0.20	(0.08, 0.52)	<0.01
Seafood consumption	High seafood consumption		27 (24.8)	48 (44.0)	0.38	(0.20, 0.72)	< 0.01
	(More than 6 meals per week)						
	Ate saltwater fish		83 (76.1)	98 (89.9)	0.35	(0.16, 0.78)	0.01
	Ate fresh water fish (Pond fish, tilapia)		47 (43.1)	60 (55.0)	0.58	(0.33, 1.04)	0.07
	Ate sardine, mackerel (Canned fish)		82 (75.2)	100 (91.7)	0.28	(0.12, 0.65)	<0.01
	Ate tuna (Canned fish)		34 (31.2)	48 (44.0)	0.53	(0.29, 0.98)	0.04
	Ate salt fish (Pickled mackerel)		76 (69.7)	99 (90.8)	0.21	(0.09, 0.50)	< 0.01
	Ate shellfish (Lobsters, crabs)		6 (5.5)	14 (12.8)	0.33	(0.11, 1.03)	0.20
	Ate shrimp		21 (19.3)	30 (27.5)	0.61	(0.31, 1.18)	0.14
Grains consumed	Rice^*g*^		106 (97.2)	108 (100)	NR*	NR*	0.99
	Fried dumpling		85 (78.0)	101 (92.7)	0.27	(0.11, 0.67)	< 0.01
	Boiled dumpling		80 (73.4)	107 (98.2)	0.07	(0.02, 0.29)	< 0.01
	White bread		54 (49.5)	84 (77.1)	0.30	(0.16, 0.57)	<0.01
	Whole wheat bread		81 (74.3)	67 (61.5)	1.74	(0.99, 3.10)	0.06
	Cakes/buns^*h*^		91 (83.5)	106 (98.1)	0.11	(0.03, 0.48)	<0.01
	Porridge, like cornmeal, oatmeal^*i*^		91 (83.5)	105 (97.2)	0.12	(0.03, 0.51)	<0.01
	Cereal^*j*^		80 (73.4)	100 (92.6)	0.28	(0.13, 0.60)	<0.01

^*^NR = Not reported because all children in the TD control group reported eating rice.

^†^If a 95% CI for the MOR does not include one, then we conclude a significant association at 5% level.

^*a*^Paternal age was missing for 4 cases and 9 controls.

^*b*^Maternal age was missing for 5 controls.

^*c*^Parental education levels were missing for 3 cases and 7 controls.

^*d*^Source of drinking water was missing for 1 control.

^*e*^Source of water for cooking was missing for 1 control.

^*f*^Fruits and vegetables consumption was missing for 1 control.

^*g*^Rice consumption was missing for 1 control.

^*h*^Cakes/buns consumption was missing for 1 control.

^*i*^Porridge consumption was missing for 1 control.

^*j*^Cereal consumption was missing for 1 control.

In the process of examining potential confounders, we assessed the association of various exposure variables and BMC by GLMs. For example, we observed a significant (*P* = 0.02) association between paternal age and BMC. The results of t-tests revealed that mean BMC of TD children were 2 to 4 years of age was significantly higher than that of children 5 to 8 years of age (12.0 μg/L vs. 10.2 μg/L; *P* = 0.01). Additionally, TD children who ate fresh water fish (pond fish, tilapia) had significantly higher BMCs than TD children who did not eat such fish (11.0 μg/L vs. 9.9 μg/L; *P* = 0.03). However, the results of t-tests assessing similar associations for the ASD cases did not reveal any significant associations, except for ASD cases with higher parental education levels that had a marginally lower mean BMC (10.5 μg/L vs. 11.6 μg/L; *P* = 0.06), compared with ASD cases whose parents both had education up to high school. Associations of exposure variables and BMC are shown in Table 3.

**Table 3:** Factors associated with blood manganese concentrations (BMC) by ASD case status and combined samples using General Linear Model (GLM) for all participants (218 children or 109 pairs)

**Exposure variables**	**Category**		**t-test**						**Univariable analysis GLM**
			**ASD cases**			**TD Controls**			
			**Mean BMC (SD) (μg/L)**		***P*-value**	**Mean BMC (SD) (μg/L)**		***P*-value**	*P*-value^***^
			Yes^*^	No^**^		Yes^*^	No^**^		
Age of Child	More than 4 years		10.8 (2.8)	11.1 (3.2)	0.64	10.2 (2.4)	12.0 (3.4)	0.01	0.29
Sex of Child	Male		10.8 (2.9)	11.2 (3.1)	0.58	10.4 (2.5)	11.1 (3.4)	0.32	0.29
Place of child’s birth	Kingston parish		11.7 (2.5)	10.7 (2.9)	0.31	10.3 (2.7)	10.8 (2.8)	0.43	0.71
Paternal age (at child’s birth)	More than 35 years		10.8 (2.7)	10.8 (3.10)	0.97	11.0 (3.5)	10.3 (2.4)	0.24	0.02
Maternal age	More than 35 years		11.0 (3.1)	10.9 (2.8)	0.85	9.9 (1.6)	10.6 (2.8)	0.45	0.53
(at child’s birth)									
Parental education levels	At least one of the parents had education beyond high school		10.5 (2.8)	11.6 (3.0)	0.06	10.7 (3.0)	10.3 (2.4)	0.38	0.82
(at child’s birth)									
Source of drinking water	Piped water		10.8 (2.9)	11.8 (1.5)	0.43	10.6 (2.7)	8.6 (1.6)	0.14	0.33
Source of cooking water	Piped water		10.8 (2.9)	11.8 (1.5)	0.43	10.6 (2.7)	8.6 (1.6)	0.14	0.37
Fruits and vegetables consumption	Root vegetables	A. Yam, sweet potato, or dasheen	10.9 (2.9)	10.9 (3.3)	0.89	10.3 (2.6)	11.1 (3.2)	0.28	0.06
		B. Carrot or pumpkin	10.9 (3.0)	10.7 (2.3)	0.81	10.5 (2.7)	10.5 (2.10)	0.99	0.66
	Leafy vegetables	A. Lettuce	11.1 (3.0)	10.7 (2.8)	0.49	10.3 (2.3)	10.6 (3.2)	0.67	0.94
		B. Callaloo, broccoli, or pakchoi	10.9 (3.0)	10.9 (2.7)	0.91	10.5 (2.7)	10.5 (3.0)	0.96	0.99
		C. Cabbage	10.7 (2.7)	11.3 (3.1)	0.26	10.4 (2.7)	11.1 (2.1)	0.49	0.14
	Legumes	String beans	11.1 (3.2)	10.8 (2.7)	0.66	10.1 (2.3)	10.7 (2.9)	0.23	0.32
	Fruits	Tomatoes	11.1 (2.9)	10.5 (2.8)	0.24	10.4 (2.5)	11.1 (3.4)	0.38	0.82
		Ackee	10.8 (2.7)	11.1 (3.1)	0.59	10.5 (2.7)	9.9 (2.1)	0.51	0.89
		Avocado	11.1 (2.1)	10.8 (3.1)	0.60	10.5 (2.7)	10.5 (2.6)	0.94	0.80
		Green banana	10.8 (3.0)	11.0 (2.6)	0.83	10.5 (2.8)	9.8 (1.8)	0.44	0.31
		Fried plantains	11.1 (2.7)	10.4 (3.3)	0.29	10.5 (2.7)	9.8 (2.7)	0.39	0.55
Seafood consumption	High seafood consumption (More than 6 meals per week)		10.9 (2.8)	10.9 (2.9)	0.97	10.8 (2.7)	10.2 (2.7)	0.24	0.63
	Ate salt water fish		11.1 (3.0)	10.2 (2.2)	0.17	10.5 (2.7)	10.2 (2.4)	0.75	0.09
	Ate fresh water fish (Pond fish, tilapia)		10.9 (2.6)	10.9 (3.1)	0.90	11.0 (2.8)	9.9 (2.5)	0.03	0.54
	Ate sardine, mackerel (Canned fish)		10.9 (3.0)	11.0 (2.7)	0.86	10.5 (2.7)	10.5 (2.8)	0.98	0.75
	Ate tuna (Canned fish)		11.0 (3.4)	10.8 (2.6)	0.85	10.5 (2.9)	10.5 (2.6)	0.99	0.82
	Ate salt fish (Pickled mackerel)		10.8 (3.0)	11.0 (2.6)	0.77	10.7 (2.7)	8.9 (1.8)	0.054	0.94
	Ate shellfish (Lobsters, crabs)		11.5 (2.3)	10.8 (2.9)	0.57	10.7 (2.2)	10.5 (2.8)	0.82	0.88
	Ate shrimp		11.0 (3.6)	10.9 (2.7)	0.83	11.1 (2.5)	10.3 (2.8)	0.25	0.58
Grain consumption	Rice		10.8 (2.8)	12.8 (5.4)	0.25	10.5 (2.7)	NR^✪^	NR^✪^	0.27
	Fried dumpling		11.0 (2.8)	10.6 (3.2)	0.63	10.5 (2.7)	9.9 (3.0)	0.50	0.30
	Boiled dumpling		11.1 (3.0)	10.7 (2.6)	0.61	10.6 (2.7)	7.6 (1.1)	0.12	0.64
	White bread		10.8 (2.6)	10.9 (3.2)	0.85	10.6 (2.8)	10.0 (2.5)	0.34	0.93
	Whole wheat bread		10.8 (3.0)	11.0 (2.7)	0.80	10.1 (2.5)	11.1 (2.9)	0.09	0.34
	Cakes/buns		10.7 (2.8)	11.7 (3.2)	0.21	10.8 (2.7)	9.6 (3.4)	0.65	0.20
	Porridge (cornmeal, oatmeal)		10.9 (3.0)	11.0 (2.5)	0.82	10.5 (2.7)	9.5 (0.8)	0.54	0.60
	Cereal		10.6 (2.5)	11.8 (3.7)	0.053	10.5 (2.7)	9.5 (3.8)	0.29	0.49

^*^The “Yes” column includes subjects who met the category specified in front of each exposure variable.

^**^The “No” column includes subjects who did not meet the category specified in front of each exposure variable.

^***^P-value is for testing associations of exposure variables with BMC among all children combined.

^✪^NR = Not reported because all children in the TD control group reported eating rice.

Note: All missing data are the same as those reported in the footnote of Table 2.

In our final multivariable GLM analysis, we compared mean BMC of ASD cases and TD controls after adjusting for paternal age, parental education, place of child’s birth (Kingston parish vs. other parishes), consumption of root vegetables (“yam, sweet potato, or dasheen”), “cabbage”, salt water fish, and cakes/buns. Our final multivariable models revealed that after controlling for the aforementioned potential confounders, there was no significant difference in mean BMC between ASD cases and TD controls (11.5 μg/L for ASD cases and 11.9 μg/L for TD controls; *P* = 0.48). Additional information regarding the unadjusted and adjusted mean BMC is shown in Table 4. This was also true for the CLR analysis that compared the adjusted odds of ASD among children having BMC in the second, third, and fourth quartiles with that of children in the lowest quartile [Adjusted odds ratio (AOR) = 3.82; 95% CI, 0.67-21.91; AOR = 1.48; 95% CI, 0.35-6.27; and AOR = 2.43; 95% CI, 0.43-13.59, respectively]. Additional details regarding unadjusted and adjusted MOR are provided in Table 5.

**Table 4:** Unadjusted and adjusted mean blood manganese concentrations (BMC) by ASD cases status based on GLMs and 109 1:1 matched pairs

	**Mean BMC (SD) ASD Cases (μg/L)**	**Mean BMC (SD) TD Controls (μg/L)**	***P*-value**
Unadjusted	10.9 (2.9)	10.5 (2.7)	0.29
**Adjusted**^***a***^	11.5 (2.7)*	11.9 (2.7)*	0.48

^*a*^Factors adjusted for include: paternal age, parental education, place of child’s birth (Kingston parish vs. other parishes), consumption of root vegetables (“yam, sweet potato, or dasheen”), “cabbage”, salt water fish, and cakes/buns.

*Standard deviation (SD) for adjusted model equals square root of means squared error in the final multivariable GLM.

**Table 5:** Unadjusted and adjusted matched odds ratios (MOR) for assessing associations between ASD status and quartiles of blood manganese concentrations (BMC) based on the CLR models for 109 ASD cases and their 1:1 matched TD controls

	**Quartile**	**n**	**MOR (95% CI)**	***P*-value**
Unadjusted	Q1 (1 μg/L – 8.9 μg/L)	56	Ref.	--
	Q2 (9.0 μg/L – 10.0 μg/L)	64	0.71 (0.32, 1.61)	0.42
	Q3 (10.0 μg/L – 12.0 μg/L)	54	1.05 (0.46, 2.40)	0.91
	Q4 (>12. μg/L)	44	0.49 (0.21, 1.15)	0.10
Adjusted^*a*^	Q1 (1 μg/L – 8.9 μg/L)	56	Ref.	--
	Q2 (9.0 μg/L – 10.0 μg/L)	64	3.82 (0.67, 21.91)	0.13
	Q3 (10.0 μg/L – 12.0 μg/L)	54	1.48 (0.35, 6.27)	0.60
	Q4 (>12.0 μg/L)	44	2.43 (0.43, 13.59)	0.31

^*a*^Factors adjusted for include: paternal age, parental education, place of child’s birth (Kingston parish vs. other parishes), consumption of root vegetables (“yam, sweet potato, or dasheen”), “cabbage”, salt water fish, and cakes/buns.

Ref. **=** Referent category for all matched odds ratios is the lowest quartile of blood manganese concentrations.

## Discussion

### Blood manganese concentrations and ASD

Our results do not indicate a relationship between postnatal BMC and ASD case status of Jamaican children ages 2–8 years. This lack of association between ASD status and BMC was observed in both univariable (10.9 μg/L in ASD cases vs. 10.5 μg/L in TD controls, *P* = 0.29) and multivariable (11.5 μg/L in ASD cases vs. 11.9 μg/L in TD controls, *P* = 0.48) analyses that controlled for potential confounding effects of paternal age, parental education levels, place of child’s birth (Kingston parish vs. other parishes), consumption of root vegetables (“yam, sweet potato, or dasheen”), “cabbage”, salt water fish, and cakes/buns. Our findings from univariable and multivariable CLR analyses also did not suggest any significant associations between BMC analyzed as a categorical variable and ASD (both p-values based on Wald’s chi-square test with 3 degrees of freedom >0.25). Specifically, compared with children having BMC in the lowest quartile, all of the 95% CIs for adjusted odds ratios of ASD among children having BMC in the second, third, and fourth quartiles contained 1 [AOR = 3.82; 95% CI, 0.67-21.91; AOR = 1.48; 95% CI, 0.35-6.27; and AOR = 2.43; 95% CI, 0.43-13.59, respectively]. Our findings indicate a lack of significant association between postnatal BMC and ASD, regardless of whether BMC is analyzed as a continuous or a categorical (i.e., 4 quartiles) variable.

To our knowledge, there are no previous studies using venous whole blood samples as a biomarker for manganese exposure in children with ASD, but our findings are consistent with results for manganese exposures measured using other methods. For example, a study from Canada reported similar mean red blood cell manganese concentrations in ASD cases and age-matched TD controls [407.4 nmol/L (22.4 μg/L) in ASD cases and 417.6 nmol/L (22.9 μg/L) in TD controls, *P* = 0.4)] [46]. Additionally, our results are consistent with those of Windham *et al*. [39] from San Francisco, California in which the adjusted odds of ASD were not significantly different for children with greater exposure to manganese in ambient air, when adjusted for maternal age, education, and race of the child (AOR = 1.09; 95% CI, 0.75-1.59) and compared with controls [39]. Furthermore, a study of children with ASD and their age- and sex-matched TD controls in Saudi Arabia also reported no significant association between ASD status and manganese levels in hair (0.38 mg/kg in ASD cases and 0.41 mg/kg in TD controls, *P* =0.71) or urine (7.32mcg/g in ASD cases and 4.81mcg/g in TD controls, *P* = 0.13) [44]. Although the aforementioned studies adjusted for some potential confounding factors, none adjusted for potential dietary confounding factors. On the other hand, our findings are in contrast with those of Abdullah *et al*. [40] who reported a lower (though marginally significant) level of postnatal manganese in the deciduous teeth of ASD cases compared to TD controls (1.87 ppm in cases vs. 2.91 ppm in controls, *P* = 0.08) [40]. Another study of children in Saudi Arabia reported significantly lower levels of manganese in hair of children with ASD compared to TD children [42] with no adjustment for potential dietary confounding factors. Nonetheless, we acknowledge that our biomarker, venous whole blood samples, and method of exposure assessment differ from each of the previously reported studies.

Because manganese is naturally present in all human tissues and there are a variety of factors that can influence the levels of manganese in biological samples, such as food and supplemental intake, the usefulness of biomarkers for assessment of cumulative exposure to manganese has been debated [36]. Though there are various potential biomarkers for measurement of manganese exposure, blood and urine have been frequently used to assess exposure in occupational setting [36]. Although manganese is naturally found at some level in all human hair, the potential for external exposure to manganese through air contamination and dust may overestimate concentrations measured using hair as a marker of exposure [36]. Manganese can also accumulate in bone and teeth [36], but differences in techniques for sampling and assessment of levels may influence results [40]. Though blood may not be a good indicator of exposure via inhalation [61], it is considered as an adequate indicator for dietary intake [62]. For this reason, we believe our results are more indicative of the influence of orally ingested manganese on ASD case status.

### Blood manganese concentration in Jamaican children

To our knowledge, there are no previously published reports on blood, hair, or urine manganese levels in Jamaican children. The mean BMC for our sample of Jamaican TD children was 10.5 μg/L (SD = 2.70 μg/L), which is lower than levels reported in other less developed countries. For example, the mean BMC for 303 children ages 8 to 11 years enrolled in the Health Effects of Arsenic Longitudinal Study (HEALS) in Araihazar, Bangladesh was 14.78 μg/L (SD = 3.72 μg/L) [18], while the mean BMC for male and female controls ages 3 to 7 years in a study from Pakistan were 29.5 μg/L (SD = 7.5 μg/L) and 31.2 μg/L (SD = 5.3 μg/L), respectively [63]. On the other hand, the mean BMC of our sample was similar to that reported from studies in emerging countries, such as South Africa, where the mean BMC in a study of children ages 9 to 13 years in Durban, South Africa was 10.1 μg/L (SD = 3.4 μg/L) [64] and was 9.80 μg/L (SD = 3.59 μg/L) in first graders enrolled in a separate study in Johannesburg, South Africa [65]. Reported mean BMC from more developed countries are close to that of our sample. For example, mean BMC for 57 female and 56 male children ages 0.29–2.4 years in Sydney, Australia, were 12.3 μg/L (SD = 4.8 μg/L) and 12.2 μg/L(SD = 6.0 μg/L), respectively [66], while the mean BMC for 38 children ages 7–9 years from the Community Actively Researching Exposure Study (CARES) in Marietta, Ohio, was 9.5 μg/L (SD = 2.4 μg/L) [67]. In September 2013, the Centers for Disease Control and Prevention (CDC) published data from NHANES 2011–2012 reporting that manganese concentrations ranged from 1.61 μg/L to 62.51 μg/L for individuals who participated in this study with ages ranging from 1 to 80 years [68]. A subgroup analysis of these publicly available data revealed a mean BMC of 10.7 μg/L (SD = 6.98 μg/L) for children between 2–8 years living in the US. Our findings indicate that BMC in TD Jamaican children are similar to those of the developed countries and much lower than those reported from some of the aforementioned developing countries.

Although the mean BMC in Jamaican children are similar to those of children living in the US, it is important to emphasize that higher levels of manganese have been shown to have adverse effects on cognitive [13] and neuromuscular function [12] in adults. More alarming are the adverse events seen in children exposed to excessive manganese including increased behavioral problems [14,15], reduced verbal and full-scale IQ [17-21]. In particular, Takser *et al*. [22] assessed psychomotor development of children in three different age groups (9 months, 3 years, and 6 years). They showed a relationship between high cord BMC and decreased attention in children 3 years of age (partial correlation r = −0.33, *P* < 0.001) after adjustment for potential confounding including child’s gender and mother’s educational level [22]. A study in 1-year old children from Mexico City reported an inverted U-shaped association between BMC and neurodevelopment, with lower Mental Development Index scores found for children in the lowest and the highest quintiles compared with children in the middle three quintiles after adjustment for several potential confounding variables [25]. Considering the potential for numerous adverse outcomes related to excessive manganese exposure, in the following we discuss factors associated with BMC in TD Jamaican children.

### Age and sex of children and their association with blood manganese concentrations

It has been reported that BMC declines with age [69-73]. For example, Claus Henn *et al*. [73] reported mean BMC of 24.7 μg/L and 21.5 μg/L for Mexican children at age 12 months and 24 months, respectively [73] and it has been shown that this decline in BMC over time is statistically significant [β = −5.7; 95% CI: −6.2 to −5.1] [26]. Our findings indicated that TD Jamaican children older than 4 years have a significantly lower mean BMC than children whose age was between 2–4 years, (10.2 μg/L vs. 12.0 μg/L, *P* = 0.01). This finding is consistent with evidence of an inverse association between manganese and age [69-73]. This finding along with the information reported in the literature suggests that age could be a potential confounder when an association between BMC and ASD is investigated. We also did not find sex differences for BMC in our sample. These results are consistent with those of Clause Henn *et al.*[73] who also reported no sex differences in BMC. Additionally, a study in TD Pakistani children age 3 to 7 years reported a mean BMC of 29.5 μg/L (SD = 7.5 μg/L) in 104 boys and 31.2 μg/L (SD = 5.3 μg/L) in 106 girls [63]; although a formal comparison by sex is not reported in this paper, our calculation of a z-score for the mean difference based on the summary data reported resulted in Z = 1.89 with a two-side p-value of 0.07, suggesting a borderline, but not significant, association between sex and BMC. In our study since we matched ASD cases and TD controls by age and sex in the study design phase, we did not include age and sex in our multivariable models that investigated the possible association between BMC and ASD.

### Seafood consumption and blood manganese concentrations

Residents of islands often consume larger amounts of seafood compared to those living in other habitats. Through contaminated water, manganese can accumulate in tissues of fish and other sea creatures [32-34,74,75]. Manganese seems to accumulate in certain fish species with considerable biological significance [75]. For example, Kumar *et al*. [76] reported that manganese levels in aquaculture ponds fish were 2.4 times higher than in coastal water fish. Our analysis indicated that TD children who ate fresh water fish (pond fish, tilapia) had a higher BMC than children who did not (11.0 μg/L vs. 9.9 μg/L; *P* = 0.03). Considering that results from a study of heavy metal concentrations in seafood purchased in a coastal community in Canada showed that metal concentrations, including manganese, in locally caught seafood products were higher than that observed in imported products [34] and another in New Jersey reported correlations between manganese concentrations in certain seafood products (fish tissue) [74] these findings are not surprising. Though the risks and benefits of eating fish and seafood have been debated, especially concerning exposure to heavy metals [77,78], our results indicate that there is a significant increased risk of manganese exposure *via* fish consumption in Jamaican children.

### Limitations

Since potential ASD cases in the UWI Jamaica Autism Database are referred from all over the island and TD controls were more likely to be from the Kingston area, a higher number of the TD controls are born in Kingston parish than the ASD cases. Therefore, manganese concentrations from our TD controls may not be generalizable to all children in Jamaica. In future studies it will be important to sample TD controls more widely from the island. Furthermore, although we used a culturally sensitive food frequency questionnaire that had been previously utilized in Jamaica, there is potential for recall bias from respondents. Although we used blood manganese concentrations as our biomarker of exposure, we acknowledge that tissue markers of exposure, such as nervous tissue, may be better indicators of long term exposures. Though prior studies have indicated that hair manganese levels increase over years of exposure, these measurements may be influenced by external exposures as well [79]. Considering that the best biomarker of manganese exposures is still not established, we used blood manganese concentration, which is considered as an adequate biomarker for body burden [79,80]. Our data collection method also could not account for timing of manganese exposure *via* food consumption, so responses may only represent recent exposures. In addition, our BMC data were not differentiated by type of manganese exposures (i.e., organic and inorganic), so we could not provide any discussion regarding this distinction based on our data. In addition, it is important to note that BMC measurements for children represent “current” exposure, implying that the manganese exposure for ASD cases reflect BMC after the child is already diagnosed with ASD. While we did not observe a relationship between blood manganese concentrations and ASD status, we acknowledge that it is possible that prenatal or early life exposures may be more predictive of ASD. We also lack information on maternal diet or manganese levels during pregnancy. Although we did not find differences in BMC for children with ASD versus TD controls, there may have been genetic differences in susceptibility to manganese exposure. Palmer *et al*. [81] suggested that without attention to gene-environment interaction, results of tests for safety of environmental exposures, such as manganese, should not be interpreted as confirmatory [81]. Despite our initial effort to keep the age of matched TD control children within six months of ASD cases, for 12.8% of pairs the age of the TD controls was within 7–15 months. However, we do not believe this difference would be likely to have any significant impact on the findings reported here.

## Conclusions

Our study results found no statistically significant association between BMC and ASD status. To our knowledge, we are the first to report no association between blood manganese concentrations and ASD in children, while controlling for dietary confounding factors. Furthermore, this is the first study to report blood manganese levels of children residing in Jamaica. Our findings demonstrated that BMC for children in Jamaica were lower than that of less developed countries and similar to that of more developed countries.

## Competing interests

The authors declare that they have no competing interests.

## Authors’ contributions

MHR, MSV, KAL, JB, and EB have made substantial contributions to conception and study design; MSV and SSP contributed to acquisition of data; MAH, SSP, MSV, MLG, and MHR have made contributions to data quality assurance procedures; MHR, ASD, and MAH conducted data analysis; MHR, ASD, MAH, MSV, and JB have contributed to interpretation of data; MHR, ASD, and MAH significantly contributed to drafting of the manuscript, and MHR, KAL, MSV, MLG, DAP, JB and EB provided critical revision of the manuscript; All authors have read and approved the final version submitted for publication.
